# Benign mammary epithelial cells enhance the transformed phenotype of human breast cancer cells

**DOI:** 10.1186/1471-2407-10-373

**Published:** 2010-07-16

**Authors:** Joanna M Poczobutt, John Tentler, Xian Lu, Pepper J Schedin, Arthur Gutierrez-Hartmann

**Affiliations:** 1 Molecular Biology Program, University of Colorado Denver, Aurora, CO 80045, USA; 2 Department of Medicine, University of Colorado Denver, Aurora, CO 80045, USA; 3 Department of Biochemistry and Molecular Genetics, University of Colorado Denver, Aurora, CO 80045, USA; 4 University of Colorado Cancer Center Biostatistics and Bioinformatics Core, University of Colorado Denver, Aurora, CO 80045, USA

## Abstract

**Background:**

Recent research has yielded a wealth of data underscoring the key role of the cancer microenvironment, especially immune and stromal cells, in the progression of cancer and the development of metastases. However, the role of adjacent benign epithelial cells, which provide initial cell-cell contacts with cancer cells, in tumor progression has not been thoroughly examined. In this report we addressed the question whether benign MECs alter the transformed phenotype of human breast cancer cells.

**Methods:**

We used both *in vitro *and *in vivo *co-cultivation approaches, whereby we mixed GFP-tagged MCF-10A cells (G2B-10A), as a model of benign mammary epithelial cells (MECs), and RFP-tagged MDA-MB-231-TIAS cells (R2-T1AS), as a model of breast cancer cells.

**Results:**

The *in vitro *studies showed that G2B-10A cells increase the colony formation of R2-T1AS cells in both soft agar and clonogenicity assays. Conditioned media derived from G2B-10A cells enhanced colony formation of R2-T1AS cells, whereas prior paraformaldehyde (PFA) fixation of G2B-10A cells abrogated this enhancement effect. Moreover, two other models of benign MECs, MCF-12A and HuMECs, also enhanced R2-T1AS colony growth in soft agar and clonogenicity assays. These data reveal that factors secreted by benign MECs are responsible for the observed enhancement of the R2-T1AS transformed phenotype. To determine whether G2B-10A cells enhance the tumorigenic growth of co-injected R2-T1AS cells *in vivo*, we used the nude mouse xenograft assay. Co-injecting R2-T1AS cells with G2B-10A cells ± PFA-fixation, revealed that G2B-10A cells promoted a ~3-fold increase in tumor growth, irrespective of PFA pre-treatment. These results indicate that soluble factors secreted by G2B-10A cells play a less important role in promoting R2-T1AS tumorigenesis *in vivo*, and that additional components are operative in the nude mouse xenograft assay. Finally, using array analysis, we found that both live and PFA-fixed G2B-10A cells induced R2-T1AS cells to secrete specific cytokines (IL-6 and GM-CSF), suggesting that cell-cell contact activates R2-T1AS cells.

**Conclusions:**

Taken together, these data shift our understanding of adjacent benign epithelial cells in the cancer process, from passive, noncontributory cells to an active and tumor-promoting vicinal cell population that may have significant effects early, when benign cells outnumber malignant cells.

## Background

In recent years it has become increasingly clear that the interactions between neoplastic cells and their tissue microenvironment have a profound role in the progress of cancer. Strong support for this concept comes from epidemiologic studies, which have linked several inflammatory conditions with an increased risk of cancer [1-3]. Furthermore, pregnancy-associated breast cancer diagnosed in the post-partum period is characterized by a high incidence of metastases, which have been proposed to be due to the pro-inflammatory changes in the mammary gland that occur during involution [4-6]. Thus, both pathologic and physiologic inflammatory conditions appear to contribute to tumor pathology by creating a microenvironment conducive to the progression and spread of cancer. In addition to epidemiological data, the important role of the microenvironment in cancer has been underscored by multiple *in vitro *and *in vivo *studies. For example, it has been well documented that most primary and metastatic tumors are infiltrated by immune cells that produce cytokines, cytotoxic mediators, interleukins, interferons, proteases, growth factors, and angiogenic and lymphangiogenic factors, all of which can be co-opted by neoplastic cells to contribute to the progression of cancer and development of metastases [1,2,7]. A large body of evidence exists documenting the supporting role of stromal cells in the progression of cancer. *In vivo *studies, which used co-injections of cancer cells with activated stromal cells in nude mouse xenografts, transgenic mouse model with fibroblast-specific knock-out of TRIIβ (TGF receptor IIβ), or irradiation of murine mammary fat pad, have consistently demonstrated that activated stromal cells have a profound enhancing effect on the growth of tumors, progression to a more aggressive phenotype, and formation of metastases [8-14]. These studies also led to identification of SDF-1 and CCL5 as the cytokines mediating the interactions between stromal cells and breast cancer cells [11,14]. *In vitro *studies have similarly shown that stromal cells were able to induce growth-factor expression, as well as promote proliferation and invasion of cancer cells [12,14,15]. While an abnormal microenvironment can promote the tumorigenic process, the normal microenvironment may be able to suppress it, as has been demonstrated by normal myoepithelial cells inhibiting the growth and progression of breast carcinoma cells in a nude mouse xenograft [10]. Furthermore, the oncogenic potential of transformed cells can be suppressed by a normal embryonic environment [16-18].

As illustrated by the examples above, the main research efforts have concentrated on delineating the interactions between cancer cells and their surrounding stroma. However, cancer cells may also interact with the vicinal normal epithelial cells or with non-neoplastic epithelial cells that have acquired some mutations but have remained benign. While in advanced tumors non-neoplastic epithelial cells may comprise a minute fraction of the tumor microenvironment, in small, early lesions, such as ductal carcinoma in situ of the breast, the ratio of normal or benign epithelial cells to cancer cells may favor the interaction with these epithelial cells as critical determinants of cancer cell fate. Indeed, direct contacts between microscopic malignant lesions and normal appearing epithelial cells have been observed in breast and in prostate tissues, however the biological effects of these interactions are unknown [19,20]. Such interactions may determine whether cancer cells undergo apoptosis, become quiescent, or advance to clinically-relevant tumors. Thus, the various modes by which the non-neoplastic mammary epithelial cells may control the fate of early neoplastic lesions provide important considerations for strategies for cancer treatment and chemoprevention. Despite their potential importance, the interactions between normal or benign epithelial cells and cancer cells have not been examined in detail. The few published *in vitro *studies have used various models of normal mammary epithelial cells to demonstrate both inhibitory effects of normal epithelial cells on cancer cells, such as inhibition of breast cancer cell proliferation, and stimulatory effects, such as enhancement of breast cancer cell invasion, with the latter being dependent on SDF-1 [21-26]. However, the precise mechanism by which normal or benign epithelial cells influence the transformed phenotype of cancer cells is not known.

In this study, we used both *in vitro *and *in vivo *approaches to address the hypothesis that benign mammary epithelial cells influence the transformed phenotype of breast cancer cells. Our data show that soluble factors secreted by benign mammary epithelial cells stimulate formation of colonies in soft agar by breast cancer cells and increase their clonogenic growth in tissue culture. We also show that G2B-10A benign mammary epithelial cells stimulate the tumorigenic growth of R2-T1AS cells in the nude mouse xenograft assay, however, this effects is not dependent on factors secreted by the benign cells, but rather may be the result of cell-cell interactions.

## Methods

### Cell lines and cell culture

MCF-10A and MCF-12A cell lines were obtained from American Type Culture Collection. G2B-10A and G-12A cell lines were generated from MCF-10A and MCF-12A cells, respectively, by infection with a GFP-expressing lentivirus, as described below. G2B-10A and G-12A cells were maintained in DMEM/F-12 medium (Invitrogen, #11330) supplemented with 10 μg/ml insulin (Sigma, #I5500), 0.5 μg/ml hydrocortisone (Sigma, #H0888), 100 ng/ml cholera toxin (Sigma, #C8052), 20 ng/ml EGF (epidermal growth factor receptor, Invitrogen, #53003-018), and 5% horse serum (Invitrogen, #16050-122). Cells were passaged with trypsin twice a week. HuMEC cells (a kind gift from Dr. DeGregori, University of Colorado) are human mammary epithelial cells immortalized with telomerase. HuMEC cells were maintained in HuMEC basal serum-free medium (Invitrogen, # 12753018) supplemented with HuMEC supplement kit (Invitrogen, # 12755013), and passaged with trypsin three times a week, according to the media manufacturer's protocol. MDA-MB-231-T1AS cells are a variant of MDA-MB-231 selected for high tumorigenicity in mice [27]. The red-marked R2-T1AS cells were generated from MDA-MB-231-T1AS cells by infection with DsRed2-expressing lentivirus, as described below. R2-T1AS cells were cultured in high-glucose DMEM medium (Invitrogen, #11965) supplemented with 15% horse serum (Invitrogen, #16050-122), 2.5% fetal bovine serum (FBS, Invitrogen, #16000-044), and non-essential amino-acids (Invitrogen, #11140-050). Cells were passaged with trypsin three times a week.

### Lentivirus production and infection of target cells

The lentiviral vectors FUW (empty vector) and FUGW (GFP-expressing) [28] were kindly provided by Dr. DeGregori. To generate FURW (DsRed2-expressing) lentivirus we subcloned the cDNA encoding DsRed2 red fluorescent protein into FUW lentiviral backbone. To this end, we digested pDsRed2-N1 plasmid (Clontech, kindly provided by Dr. Verkhusha, University of Colorado Denver) with BamHI and MfeI restriction enzymes and inserted the resulting 800 bp fragment into FUW vector, which was digested with BamHI and EcoRI. Virus-containing supernatant was generated by Effectene-mediated (Qiagen, #301425) co-transfection of 293T fibroblasts with the following plasmids: (1) FUGW or FURW viral gene-transfer vector, (2) Delta8.9, the HIV-1 packaging plasmid, expressing gag-pol and accessory proteins, and (3) pCMV-VSVg, the envelope glycoprotein plasmid. Virus-containing supernatant was collected 48 and 72 h post-transfection, filtered through 0.45 μm syringe filter and stored at -80°C. To infect cells, the virus-containing supernatant was diluted in growth medium 1:3, supplemented with polybrene (8 μg/ml), and overlaid on the target cells. After overnight incubation with the viral supernatant, medium was changed to fresh culture medium. Expression of EGPF or DsRed2 was detectable by fluorescence microscopy after 48-72 h post infection.

### Paraformaldehyde (PFA) fixation of G2B-10A cells

G2B-10A cells were harvested with trypsin, and resuspended in culture medium. An equal volume of 4% PFA solution was added to the cell suspension, resulting in the final PFA concentration of 2%. The cells were fixed for 20 minutes at room temperature, and washed three times with 50 ml of phosphate-buffered saline (PBS).

### Soft agar assays

Soft agar assays were performed in 6-well plates, in assay medium containing 0.25 g/L glucose (no-glucose DMEM, Invitrogen, #11966 plus low-glucose DMEM, Invitrogen#11885, mixed 3:1), supplemented with 100 mM sodium pyruvate (Invitrogen, #11360) and 5% FBS. Individual wells were coated with 1.5 ml of base medium composed of the aforementioned assay medium mixed with 1% agar (BD, #214230) stock to yield a final concentration of 0.6% agar. The cells (R2-T1AS, G2B-10A, G-12A, HuMEC) were harvested with trypsin, resuspended and mixed in assay medium at various densities and ratios, according to each experimental design. Volumes of cell suspensions were adjusted with the assay medium to be equal for all conditions. In all soft agar cultures, R2-T1AS cells were plated at a density 1 × 10^4 ^cells/well. G2B-10A, G-12A and HuMEC cells were admixed in co-cultures at densities 1 × 10^4 ^to 8 × 10^4 ^cells/well. All cell suspensions were mixed with the aforementioned assay medium and 1% agar stock, so that the final concentration of agar was 0.3%, and immediately plated on solidified base layers in a 1.5 ml volume. Soft agar cultures were incubated for 21 days and fed with 150 μl of the assay medium twice a week. The resulting colonies were treated with 200 μl Nitroblue reagent (1 mg/ml, Amresco, #0329) and incubated at 37°C overnight to develop a blue stain. Colonies were photographed with a digital camera (Kodak) and the acquired images were analyzed using the ImageJ software. Colonies larger than 150 μm in diameter were scored as positive.

Soft agar assays with conditioned media were conducted using the same protocol as above, except the assay medium was mixed with the conditioned media at a 1:1 ratio.

### Conditioned media preparation

To generate conditioned media for the soft agar assay, cells were plated on 10 cm tissue culture plates in 10 ml of 0.25 g/L glucose, 5% FBS medium (see soft agar assays). Cell cultures were set up as follows: G2B-10A-conditioned medium - 6 × 10^6 ^of G2B cells per plate; G2B-10A(PFA)-conditioned medium 6 × 10^6 ^G2B-10A(PFA) cells per plate. Medium alone ("mock") was included as a negative control.

To generate conditioned media for clonogenic growth assay, cells (G2B-10A, G-12A or HuMEC) were plated in their respective culture media on 10 cm tissue culture plates at a density of 3 or 6 × 10^6 ^cells/plate. After overnight incubation the cells were washed once and media were changed to 10 ml of 0.25 g/l glucose media not supplemented with FBS. Medium alone ("mock") was included as a negative control. These media were supplemented with 5% FBS prior to the clonogenicity assay.

To generate conditioned media for analysis by cytokine antibody array cell cultures were set up in suspension, on 6-cm plates coated with soft agar base layers (0.25 g/l glucose, 5% FBS medium containing 0.6% agar). Cells were suspended in 4 ml of 0.25 g/l glucose, 5% FBS medium and plated on solidified base layers. Cell cultures were set up as follows: R2-T1AS-only - 0.3 × 10^6 ^R2-T1AS cells per plate, G2B-10A-only - 2.4 × 10^6 ^of G2B cells per plate, R2-T1AS/G2B-10A - 0.3 × 10^6 ^R2-T1AS plus 2.4 × 10^6 ^of G2B cells per plate, R2-T1AS/G2B-10A(PFA) - 0.3 × 10^6 ^R2-T1AS plus 2.4 × 10^6 ^G2B-10A(PFA) cells per plate.

After 3 days of incubation at 37°C, conditioned media were harvested, centrifuged, filtered through 0.45 μm syringe filter to remove cells and debris, and stored at 4°C or frozen at -80°C, until needed.

### Proliferation in suspension assay

The R2-T1AS cells were harvested with trypsin, resuspended in 0.25 g/l glucose, 5% FBS medium (see soft agar assays) and seeded in 6-well, low-attachment plates (Corning, #3471) at a density of 3 × 10^5 ^cells/well in 2 ml volume. Subsequently, an equal volume of conditioned medium (prepared as for soft agar assays) was added to the wells. After 24, 48, and 96 hours of incubation cells were harvested, triturated to dislodge any cell aggregates, and counted using an automated cell counter (Vi-cell XR, Coulter). Viability of the cells was determined using trypan blue exclusion.

### Clonogenic growth assay

The R2-T1AS cells were harvested with trypsin, resuspended in 0.25 g/l glucose, 5% FBS medium (see soft agar assays) and plated in 24-well tissue culture plate at a density 40 cells/well in a 150 μl volume. Subsequently, an equal volume of conditioned medium, supplemented with 5% FBS, was added to the wells. The cells were incubated at 37°C for 5 days, fixed in 2% PFA and stained with Hoechst dye to visualize the nuclei. Colonies were counted under the fluorescent microscope and those larger than 10 cells were scored as positive.

### Antibody array

The conditioned media were analyzed using cytokine antibody array (RayBiotech, Human Cytokine Antibody Array 3) according to manufacturer's protocol. Briefly, membranes were blocked for 2 h, afterwards, 1 ml of conditioned media was added to the membranes and incubated at 4°C overnight. Detection antibody cocktail was incubated for 0.5 h, followed by HRP-conjugated streptavidin incubation for 0.5 h. Signal was detected on an X-ray film using the reagents provided by the manufacturer. The arrays were quantified using ImageJ. The two signals for each cytokine were averaged and normalized to the average of the six positive control signals on each array.

### ELISA

The conditioned media were analyzed using ELISA kits for the detection of human IL-6 (RayBiotech, #ELH-IL6-001) and human MCP-1 (RayBiotech, #ELH-MCP1-001). Briefly, samples of 100 μl of conditioned media were incubated on ELISA plates at 4°C overnight, followed by incubations with detection reagents as specified in the manufacturer's protocol. Signal was read at 450 nm using a BioTek Synergy HT plate reader.

### Nude mouse xenograft assay

Xenograft experiments were conducted in 7-8 week old nude female mice, purchased from the NCI. During the experiment, mice were supplemented with estrogen released from a subcutaneously placed pellet [29], which we provided in the event that the injected cells restore estrogen receptor expression and become estrogen-dependent *in vivo*. The R2-T1AS and G2B-10A cells were harvested by trypsinization and resuspended in PBS at a density 1 × 10^5 ^cells/μl. In the R2-T1AS/G2B-10A group, cells were injected as 1 × 10^6 ^R2-T1AS cells plus 4 × 10^6 ^G2B-10A cells per injection (50 μl). In the R2-T1AS/G2B-10A(PFA) group, cells were injected as 1 × 10^6 ^R2-T1AS cells mixed with 4 × 10^6 ^PFA-fixed G2B-10A cells per injection (50 μl). In the R2-T1AS-only control, 1 × 10^6 ^R2-T1AS cells were injected alone (10 μl) and in the G2B-10A-only control 4 × 10^6 ^G2B-10A cells were injected alone (40 μl). Cells were injected bilaterally onto mammary fat pads #4. Tumor size was assessed on a weekly basis by measurements with an electronic caliper. Volume was calculated as 0.52 × length × width^2^. Nude mouse xenograft experiments were performed under an animal protocol approved by the Animal Care and Use Committee of the University of Colorado Denver. Data shown are combined from 2 independent experiments, with 4 injections completed for the G2B-10A-only group, 10 injections for the R2-T1AS-only group, 6 injections for the R2-T1AS/G2B-10A group, and 10 injections for the R2-T1AS/G2B-10A(PFA) group.

### Tissue processing

Tumors were harvested at day 7 and 14 after inoculation and immediately fixed in 4% PFA in PBS at room temperature, overnight. Fixed tumors were subsequently cryopreserved for 24h in 30% sucrose in PBS at 4°C. Thereafter, tissues were embedded in Tissue-Tek O.C.T. medium (Sakura, #4583), frozen on dry ice, and stored at -80°C. For microscopic analysis, frozen tumors were cut into 10 μm sections and placed on glass slides.

### Fluorescence analysis

Sections were thawed at room temperature, washed with PBS and counterstained with Hoechst dye (Sigma) for 5 min to visualize the nuclei. Slides were mounted using Fluoromount-G medium (Fisher) and imaged using fluorescent microscopy. Shown images are composites of several low-magnification (4×) pictures.

### Statistical analyses

Statistical analyses were performed by the University of Colorado Cancer Center Biostatistics and Bioinformatics Core. Data were analyzed using linear regression models for colony numbers and linear mixed models for tumor volume (mm^3^) over time. Least squares means were estimated and compared, and p-values were adjusted with Dunnett or Tukey-Kramer post-hoc adjustment methods for multiple comparisons. Tukey's method is appropriate when all pairwise comparisons are performed at once, while Dunnett's method is appropriate for pairwise comparisons between experimental and control groups only. The family-wise error rate was fixed at 0.05. Because of the observed skewness in tumor volume data, we also performed linear mixed model analyses on the natural log transformed values (log mm^3^). The conclusions from these analyses were the same as those using raw tumor volumes. Only the latter results are presented. The data analyses for this paper were generated using SAS/STAT software, Version 9.2 of the SAS System for Windows. Copyright^© ^2002-2008 SAS Institute Inc. SAS and all other SAS Institute Inc. product or service names are registered trademarks or trademarks of SAS Institute Inc., Cary, NC, USA.

## Results

### Generation of G2B-10A and R2-T1AS cells as models of benign mammary epithelial cells and breast cancer cells, respectively

In this study we addressed the hypothesis that benign mammary epithelial cells influence the transformed phenotype of breast cancer cells. As our primary model of benign mammary epithelial cells we chose the non-transformed MCF-10A human mammary epithelial cell line. These cells are spontaneously immortalized, however they exhibit multiple characteristics of benign mammary epithelium, such as growth factor-dependent proliferation, lack of anchorage-independent growth, and lack of tumorigenicity in nude mice [30]. In order to track the benign MCF-10A cells in subsequent studies, we marked them with green fluorescent protein (GFP) by lentiviral transduction to generate the G2B-10A line. We confirmed that G2B-10A cells retained their benign phenotype by showing that they maintained the ability to form acini in the 3-dimensional matrigel assay (not shown), as is typical for benign mammary epithelial cells [31,32]. As additional models of benign mammary epithelial cells, we used HuMEC cells, which are human primary mammary epithelial cells immortalized by expression of telomerase, and MCF-12A cells, which we also marked with GFP to generate the G-12A line [33]. As a model of breast cancer, we chose MDA-MB-231-T1AS cell line, which is a derivative of MDA-MB-231 cells, selected for high tumorigenicity in mice by *in vivo *selection [27]. We marked these cells with red fluorescent protein (DsRed2) by lentiviral transduction and denoted them R2-T1AS breast cancer cells.

### G2B-10A benign mammary epithelial cells enhance the anchorage-independent growth of R2-T1AS breast cancer cells in soft agar

**Figure 1:**
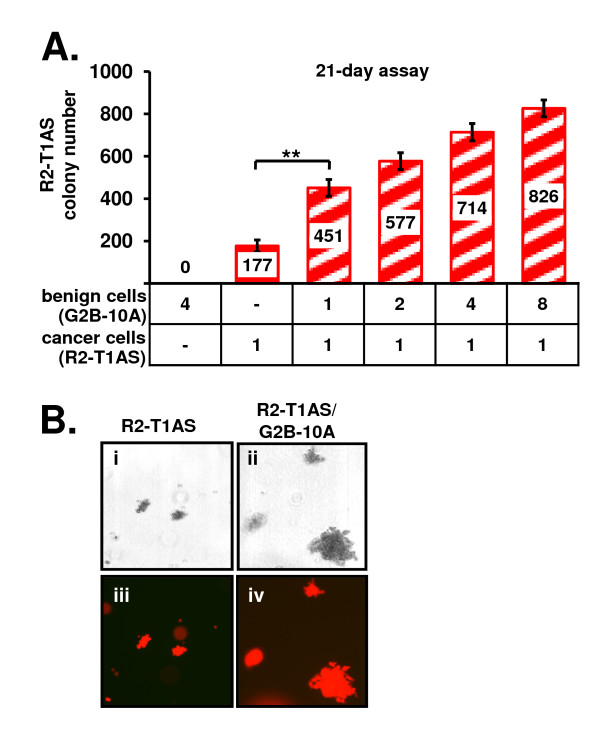
**G2B-10A benign mammary epithelial cells enhance the anchorage-independent growth of R2-T1AS breast cancer cells in soft agar**. (A) Soft agar co-culture of R2-T1AS cells and G2B-10A cells. G2B-10A cells and R2-T1AS were mixed at the indicated ratios and plated in soft agar cultures in low-glucose, 5% serum media. Colony numbers were scored after 21-day incubation. R2-T1AS cells were plated at a density of 1 × 10^4 ^cells/well in each culture (denoted as 1 in the graph labels), and G2B-10A cells were admixed at 1 × 10^4 ^to 8 × 10^4 ^cells/well, as indicated (denoted as 1 to 8 in the graph labels). Red bar - R2-T1AS plated alone; red stippled bars - R2-T1AS plated with G2B-10A cells. Graph shows mean number of R2-T1AS colonies (± standard error) obtained in 4 independent experiments, with each experiment performed in 4 replicate wells. ** p < 0.001 (B) Soft agar colonies are composed exclusively of R2-T1AS cells. Brightfield (panels i & ii) and fluorescent images (panels iii & iv) of colonies formed after 21-day incubation. Panels i & iii: The R2-T1AS-only culture; Panels ii & iv: R2-T1AS/G2B-10A co-culture containing exclusively colonies formed by red-marked R2-T1AS cells (red).

The ability to survive and proliferate without attachment to substratum is a feature associated with neoplastic transformation. To examine how this anchorage-independent survival and growth of R2-T1AS breast cancer cells is modulated by the interactions with benign mammary epithelial cells, we used the soft agar colony formation assay. The soft agar assays were carried out for 21 days in low glucose, 5% serum soft agar medium. We found that in lower serum concentrations, cancer cells failed to form colonies in soft agar. Thus, the 5% serum conditions were optimal to study the effects of benign cells on cancer cells. To control for the effects of the factors present in serum, we used medium containing 5% serum in negative control cultures in all our experiments. First, we verified that the benign G2B-10A cells do not form colonies in soft agar, by plating 4 × 10^4 ^G2B-10A cells alone, and indeed, we did not detect any colonies in this culture (Figure 1A). To establish the soft-agar colony number formed by R2-T1AS cells, we plated 1 × 10^4 ^R2-T1AS cells alone, which yielded 177 colonies (Figure 1A, red bar). To determine whether G2B-10A cells influence the R2-T1AS-colony formation, we co-cultured 1 × 10^4 ^R2-T1AS cells with G2B-10A cells, from 1 × 10^4 ^to 8 × 10^4 ^cells, resulting in increasing cancer cell to benign MEC ratios from 1:1 to 1:8. In the co-culture at a 1:1 ratio, we detected 451 colonies, which represents a 2.5-fold increase over the R2-T1AS-only culture. The number of colonies increased with the number of G2B-10A cells admixed, reaching to 826 for the 1:8 ratio (Figure 1A, red stippled bars). Thus, the G2B-10A benign mammary epithelial cells increased the soft-agar colony formation of R2-T1AS breast cancer cells in a manner dependent on the number of G2B-10A cells admixed.

### Soft agar colonies are composed exclusively of R2-T1AS cells

To determine whether the benign mammary epithelial cells contributed to soft agar colonies, we analyzed the co-cultures using brightfield and fluorescence microscopy. Figure 1B shows brightfield images (Figure 1B panels i & ii) and the corresponding fluorescent pictures (Figure 1B, panels iii & iv) of the colonies formed after a 21-day incubation in the R2-T1AS-only culture (Figure 1B, panels i & iii) and in the R2-T1AS/G2B-10A co-culture (Figure 1B, panels ii & iv). As expected, all the colonies visualized in the R2-T1AS-only culture were composed of red-fluorescent R2-T1AS cells. In the R2-T1AS/G2B-10A co-culture, we also observed that all colonies were composed of red-marked R2-T1AS cells. We did not detect any green-marked G2B-10A cells in the R2-T1AS/G2B-10A co-culture. Almost all of the G2B-10A cells died within 7-10 days (not shown).

### G-12A and HuMEC cells increase soft agar growth of R2-T1AS cells

**Figure 2:**
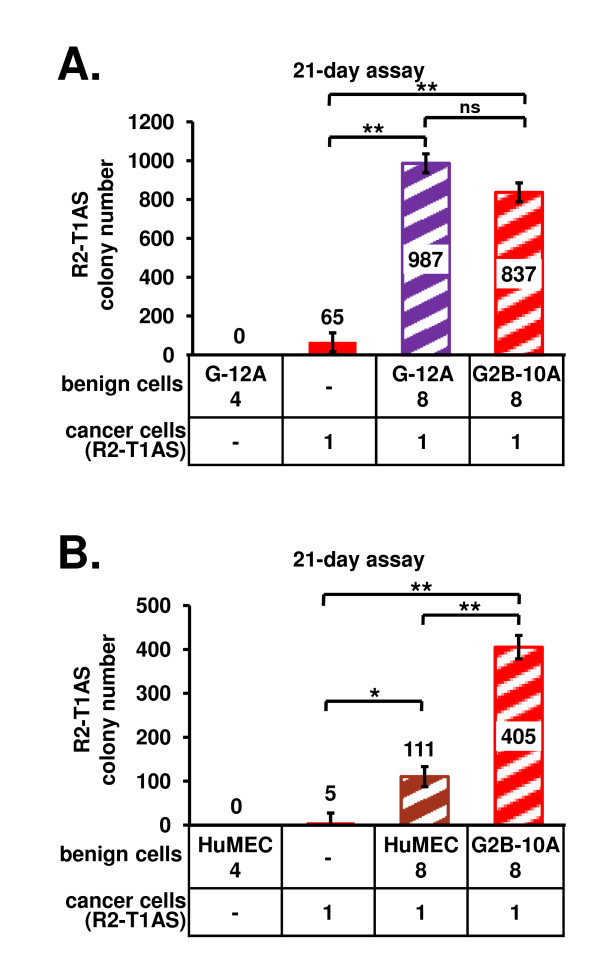
**G-12A and HuMEC benign mammary epithelial cells enhance the anchorage-independent growth of R2-T1AS breast cancer cells in soft agar**. (A) Soft agar co-culture of R2-T1AS cells and G-12A cells. R2-T1AS cells were plated alone at a density of 1 × 10^4 ^cells per well (denoted as 1, red bar), in the R2-T1AS/G-12A co-culture, 1 × 10^4 ^R2-T1AS were mixed with 8 × 10^4 ^G-12A cells (denoted as 8, purple stippled bar), and in the R2-T1AS/G2B-10A co-culture 1 × 10^4 ^R2-T1AS were mixed with 8 × 10^4 ^G2B-10A cells (denoted as 8, red stippled bar). Graph shows mean colony numbers (± standard error) obtained in 3 independent experiments, with each experiment performed in 4 replicate wells. (B) Soft agar co-culture of R2-T1AS cells and HuMEC cells. R2-T1AS cells were plated alone at a density of 1 × 10^4 ^cells per well (denoted as 1, red bar). In the R2-T1AS/HuMEC co-culture 1 × 10^4 ^R2-T1AS cells were mixed with 8 × 10^4 ^HuMEC cells (denoted as 8, brown stippled bar), and the R2-T1AS/G2B-10A co-culture was plated as in Figure 2A (red stippled bar). Graph shows mean colony numbers (± standard error) obtained in 5 independent experiments, with each experiment performed in 4 replicate wells. Different numbers for R2-T1AS-only cultures (65 in Figure 2A, and 5 in Figure 2B) reflect the experimental variability, as detailed in the text. * p < 0.05, ** p < 0.001, ns - not significant.

To investigate whether the ability to enhance R2-T1AS soft agar colony formation is shared by other benign mammary epithelial cells, we tested additional benign cell line models in this assay: G-12A (Figure 2A) and HuMEC cells (Figure 2B). We determined that G-12A benign cells did not form colonies in soft agar, while R2-T1AS cells plated alone formed 65 colonies in this study (Figure 2A, red bar). In the R2-T1AS/G-12A co-culture, we detected 987 colonies (Figure 2A, purple stippled bar), which was equivalent to the number of colonies counted in a concurrent R2-T1AS/G2B-10A co-culture (837 colonies, Figure 2A, red stippled bar). The result of this study shows that G-12A cells enhance the formation of colonies by R2-T1AS cells, to an extent comparable to G2B-10A cells. Upon examination of the soft agar colonies under fluorescence microscopy, we found that all colonies in the R2-T1AS/G-12A co-culture were formed exclusively by R2-T1AS cells as was the case with R2-T1AS/G-10A co-culture (data not shown).

Next, we tested the ability of HuMEC cells to increase the colony formation of R2-T1AS cells (Figure 2B). We found that HuMEC cells, when plated alone, did not form colonies in soft agar, while R2-T1AS cells plated alone in parallel cultures generated 5 colonies (Figure 2B, red bar). As explained below, the low number of colonies detected in this study appears to be the result of intrinsic experimental variability. Furthermore, R2-T1AS/HuMEC and R2-T1AS/G2B-10A co-cultures formed 111 (Figure 2B, brown stippled bar) and 405 colonies (Figure 2B, red stippled bar), respectively. This result shows that HuMEC cells enhanced the colony formation of R2-T1AS cells, however, their effect was about 4-fold less than that of G2B-10A cells. We did not examine the R2-T1AS/HuMEC co-cultures by fluorescence microscopy, since HuMEC cells were not marked with fluorescent protein, and could not be visualized by this method. Of note, the growth of R2-T1AS cells in soft agar fluctuated between experiments, possibly due to the variations in soft agar quality and/or conditions. This fluctuation is reflected by the different colony numbers in R2-T1AS-only cultures in the three studies shown in Figure 1A (177 colonies), Figure 2A (65 colonies), and Figure 2B (5 colonies), with each study consisting of 4 independent experiments and each of these independent experiments reflecting a mean of 4 wells. Despite these fluctuations, we observed a consistent increase in the colony formation between the R2-T1AS-only control and the co-cultures, which reproduced well within each single experiment (a minimum of a 2.5-fold increase). None of the benign cell lines ever formed colonies in soft agar.

### Soluble factors secreted by G2B-10A cells increase R2-T1AS colony formation in soft agar

**Figure 3:**
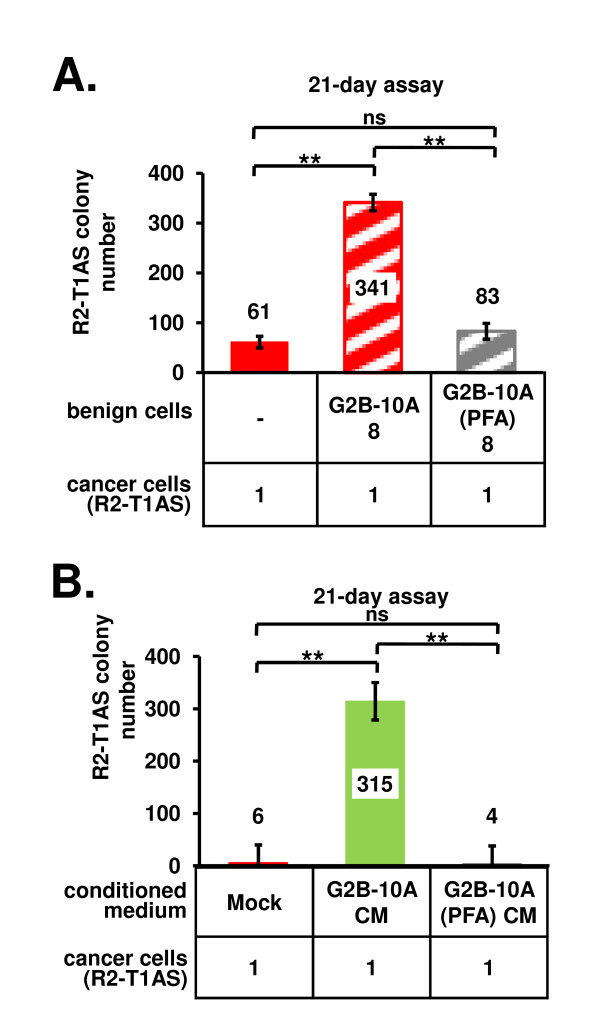
**Increased colony formation in soft agar is mediated by factors secreted by G2B-10A cells**. (A) Paraformaldehyde (PFA) fixation abrogates the ability of G2B-10A cells to stimulate R2-T1AS colony formation. As previously, 1 × 10^4 ^(denoted as 1) R2-T1AS cells, were plated in soft agar cultures either alone (red bar), or with 8 × 10^4 ^(denoted 8) live G2B-10A cells (G2B-10A, red stippled bar), or mixed with 8 × 10^4 ^(denoted as 8) PFA-fixed G2B-10A cells (G2B-10A(PFA), grey stippled bar). Data show mean numbers derived from 4 independent experiments (± standard error), with each experiment performed in 4 replicate wells. (B) Medium conditioned by G2B-10A cells enhances colony formation. To produce conditioned media, G2B-10A cells, or G2B-10A(PFA) cells, or no cells (mock) were plated in low-glucose, 5%-serum medium, on tissue culture plates. Conditioned media harvested after a 3-day incubation were used to set up the soft agar cultures. In all soft agar cultures, 1 × 10^4 ^R2-T1AS cells were plated alone (denoted as 1 in the graph labels) in either mock medium (red bar), G2B-10A - conditioned medium (G2B-10A CM, green bar), or in G2B-10A(PFA) - conditioned medium (G2B-10A(PFA) CM, grey bar). Data show mean of 3 independent assays (± standard error), with each assay performed in 4 replicate wells. **p < 0.001, ns - not significant.

Since we observed that in the soft agar assay G2B-10A cells and R2-T1AS cells did not come into physical contact (not shown), we hypothesized that the increased ability of R2-T1AS cells to form soft agar colonies was mediated by soluble factors secreted by benign G2B-10A cells into the semi-solid medium. To test this possibility, we eliminated the ability of G2B-10A to secrete factors by fixing them with paraformaldehyde (PFA), and denoted the treated cells as G2B-10A(PFA). Such cells remain intact, but are dead due to cross-linking of internal proteins, and consequently, they do not secrete any factors [34]. In this study, R2-T1AS cells formed 61 colonies when plated alone (Figure 3A, red bar) and 341 colonies when mixed with G2B-10A cells (Figure 3A, red stippled bar). When mixed with the nonsecretory G2B-10A(PFA) cells, the R2-T1AS cells yielded 83 colonies (Figure 3A, grey stippled bar), which is equivalent to the number of colonies formed by R2-T1AS alone. Thus, the increase in the number of colonies formed by R2-T1AS cells in soft agar was completely abrogated when G2B-10A cells were fixed with PFA. The results of this study, reflecting data from 4 independent experiments, with each experiment performed in 4 wells, are consistent with the idea that G2B-10A benign mammary epithelial cells support colony-formation of R2-T1AS breast cancer cells by secretion of soluble factors.

To further explore the hypothesis that soluble factors secreted by benign mammary epithelial cells mediate the enhanced colony formation by R2-T1AS cells in soft agar, we performed assays with conditioned medium derived from G2B-10A cells. To produce conditioned medium, G2B-10A cells were incubated in the same low-glucose, 5% serum medium as in the soft agar assays. As negative controls, we used medium incubated without cells (Mock) or medium incubated with G2B-10A(PFA) cells. In the "mock" medium, R2-T1AS cells formed 6 colonies (Figure 3B, red bar). This number increased to 315 for R2-T1AS cells grown in medium conditioned by G2B-10A cells (Figure 3B, green bar). However, in medium conditioned by G2B-10A(PFA) cells only 4 colonies were detected. This result confirmed that G2B-10A benign mammary epithelial cells secrete soluble factors that enhance the colony formation of R2-T1AS cells in soft agar.

### Factors secreted by benign mammary epithelial cells inhibit the proliferation of R2-T1AS cells in suspension

The fact that factors secreted by G2B-10A cells stimulated formation of colonies in soft agar by R2-T1AS cells led us to three hypotheses: (1) R2-T1AS cells are protected from anoikis; (2) the proliferation of R2-T1AS is enhanced; and/or (3) the clonogenic growth ability of R2-T1AS is enhanced. To determine whether the factors secreted by G2B-10A cells modulated the anoikis response of R2-T1AS cells, we cultured 0.3 × 10^6 ^R2-T1AS cells in suspension, in 6-well low-attachment plates in either mock-conditioned medium, or in medium conditioned by G2B-10A cells, or in medium conditioned by HuMEC cells. After 24h we counted the numbers of live R2-T1AS cells using the trypan blue exclusion method (data not shown). We did not detect any non-viable cells in any of the media conditions. This experiment shows that R2-T1AS cells are intrinsically insensitive to anoikis, and the enhanced colony formation observed with admix culture is not due to protection from anoikis.

**Figure 4:**
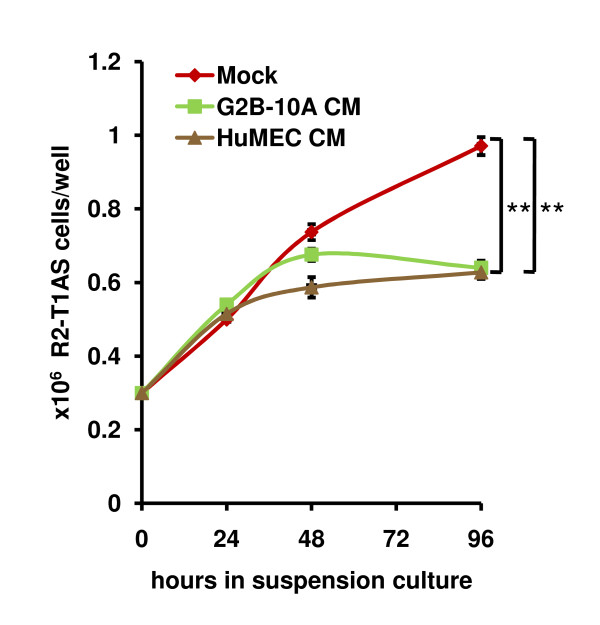
**Factors secreted by benign mammary epithelial cells inhibit the proliferation of R2-T1AS in suspension**. Graph shows proliferation of R2-T1AS cells in suspension cultures in mock-conditioned medium (red line), G2B-10A - conditioned medium (G2B-10A CM, green line), or HuMEC - conditioned medium (HuMEC CM, brown line). To produce conditioned media, G2B-10A cells, HuMEC cells, or no cells (mock) were plated in low-glucose, 5% serum medium, on tissue culture plates. Conditioned media were harvested after a 3-day incubation. For suspension culture, R2-T1AS cells were seeded in conditioned media, in 6-well, low-attachment plates, at a density of 0.3 × 10^6 ^cells per well. After 24, 48, and 96 hours media with cells were harvested and cell numbers were determined using an automated cell counter (ViCell), with the use of trypan blue exclusion. Each data point represents a mean of 3 independent experiments (± standard error), with each experiment performed in 3 replicate wells. ** p < 0.001 - for 96 hours time point.

We next determined the influence of factors secreted by benign cells on proliferation of the breast cancer cells. To this end, we cultured R2-T1AS cells in suspension in mock, G2B-10A-conditioned, or HuMEC-conditioned medium, as described above. We counted the numbers of R2-T1AS cells after 24, 48, and 96 hours of incubation (Figure 4). In mock-conditioned medium, the number of R2-T1AS cells increased over the course of the culture, yielding 0.97 × 10^6 ^cells per well at 96h (Figure 4, red line). In conditioned medium derived from G2B-10A cells we counted 0.64 × 10^6 ^cells per well (Figure 4, green line), which constitutes a 34% reduction compared to mock. In HuMEC-conditioned medium we counted 0.63 × 10^6 ^cells per well (35% reduction compared to mock), (Figure 4, brown line). Thus, the growth rate of R2-T1AS cells in conditioned media derived from both benign cell lines was slower than in mock-conditioned. This result, showing that the proliferation of R2-T1AS cells in suspension culture is inhibited by factors secreted by the benign epithelial cells, argues that a higher proliferation rate of R2-T1AS cells is unlikely to be the factor driving the increase in colony number observed in the soft agar co-cultures.

### Factors secreted by benign mammary epithelial cells stimulate clonogenic growth of R2-T1AS breast cancer cells

**Figure 5:**
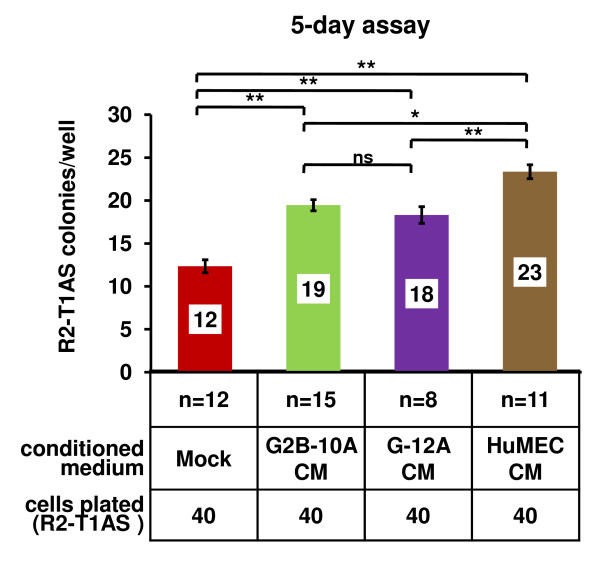
**Factors secreted by benign mammary epithelial cells stimulate clonogenic growth of R2-T1AS breast cancer cells**. Graph shows 24-well clonogenic growth assay of R2-T1AS cells in mock-conditioned medium (red bar), or in conditioned media derived from G2B-10A cells (G2B-10A CM, green bar), or G-12A cells (G-12A CM, purple bar), or HuMEC cells (HuMEC CM, brown bar). To produce conditioned media, G2B-10A, G-12A, and HuMEC cells, or no cells (mock) were cultured in serum-free, low-glucose medium for 3 days. Conditioned media were supplemented with 5% FBS at the time of the plating of R2-T1AS cells for the clonogenic growth assay. R2-T1AS cells were plated at a density of 40 cells/well in a 24-well plate, in conditioned media, as indicated. Colonies of 10 cells or more were counted after 5 days. Data represent mean number derived from 8 to 15 independent assays (denoted as "n" in graph labels), with each assay performed in 4 replicate wells. **p < 0.001, *p < 0.05, ns-not significant.

Having determined that protection from anoikis and higher proliferation rates are unlikely mechanisms responsible for the increased R2-T1AS colony number mediated by G2B-10A cells in the soft agar co-cultures, we proceeded to examine whether factors secreted by benign mammary epithelial cells stimulated the clonogenic growth of R2-T1AS cells, using conditioned media. In contrast to previous experiments, to produce conditioned media for clonogenicity assays, we cultured G2B-10A, G-12A and HuMEC cells in low-glucose medium without serum. Both mock and conditioned media were supplemented with 5% FBS before setting up the clonogenicity assays. We examined the clonogenic growth of R2-T1AS cells by plating 40 R2-T1AS cells/well in a 24-well plate in the conditioned media and scoring the number of colonies after 5 days of incubation. As shown in Figure 5, R2-T1AS cells formed 12 colonies in mock medium, 19 colonies in G2B-10A conditioned medium (58% increase compared to mock), 18 colonies in G-12A conditioned medium (50% increase), and 23 colonies in HuMEC-conditioned medium (92% increase). These data, derived from 8-15 independent experiments, with each of these experiments performed in 4 wells, demonstrate that the factors secreted by benign mammary epithelial cells stimulate clonogenic growth of R2-T1AS cells.

### G2B-10A conditioned medium contains several cytokines and specifically includes MCP-1

**Figure 6:**
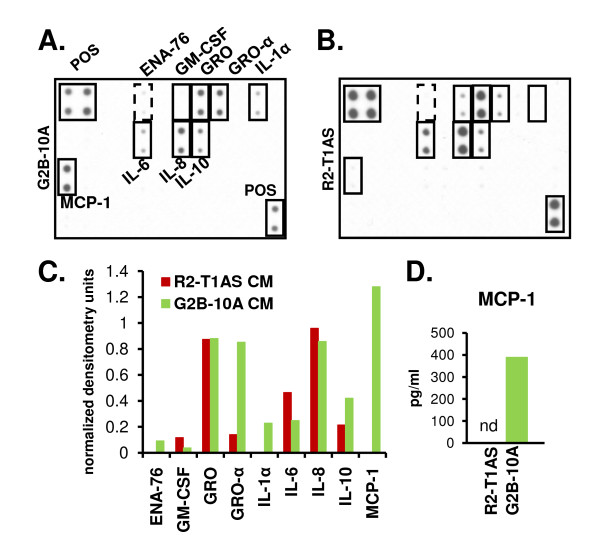
**Analysis of G2B-10A and R2-T1AS conditioned media**. (A) Cytokine antibody array analysis of medium conditioned by G2B-10A cells, plated at a density of 1.5 × 10^6 ^cells/ml. (B) Cytokine antibody array analysis of medium conditioned by R2-T1AS cells, plated at a density of 1.875 × 10^5 ^cells/ml. The R2-T1AS cells were plated at 8 times lower density to reflect the 1:8 ratio at which cancer and benign cells were co-cultured in the soft agar assays. Cells were cultured in suspension, in low-glucose, 5% serum medium. Conditioned media were harvested after 3 days of incubation and analyzed by cytokine antibody array. Both membranes were incubated with 1 ml of conditioned media derived from the indicated cell lines, underwent the same treatment and were exposed to the X-ray film for the same time. POS - positive control spots, solid lines - cytokines expressed differentially in the two cultures, dashed lines - not reproducible signals. (C) Quantification of the arrays in Figures 6A and 6B. The signals on the G2B-10A array (green bars) and R2-T1AS array (red bars) were quantified by densitometry and normalized to the average of positive control signals (POS). (D) Analysis of MCP-1 concentration in media conditioned by R2-T1AS cells or G2B-10A cells (green bar) by ELISA. nd - not detectable.

To identify factors selectively secreted by G2B-10A or R2-T1AS cells, we screened conditioned medium derived from both cell cultures, using a cytokine antibody array. The R2-T1AS cells were plated at 8 times lower cell number, to reflect the 1:8 (R2-T1AS/G2B-10A) ratio, at which they were co-cultured compared to G2B-10A cells in the soft agar assay. This way, by comparing factors secreted by G2B-10A versus R2-T1AS cells alone, we were able to establish whether any factors represented on the array were specifically secreted by one cell type or the other. To produce the conditioned media, G2B-10A and R2-T1AS cells were cultured in suspension, in low-glucose, 5% serum medium. The cytokine antibody arrays (Figures 6A &6B), which we quantified by densitometry (Figure 6C), show that a strong MCP-1 signal and a weak IL-1α signal were detected specifically in the G2B-10A-conditioned medium (Figure 6A). We also detected a weak ENA-76 signal specific to G2B-10A conditioned medium (Figure 6A), however this signal was not reproducible. Furthermore, the GRO-α signal was increased 6-fold and the IL-10 signal was increased 2-fold in the G2B-10A-conditioned medium, compared to the R2-T1AS-conditioned medium. In the repeat assay, we also detected increased GRO signal in G2B-10A conditioned medium compared to R2-T1AS medium. However, the GRO-α signals were equal in the study shown in Figures 6A and 6B. In the R2-T1AS media, we detected IL-6 and GM-CSF signals that were weakly detectable on the G2B-10A array (Figure 6A and 6B). Both media generated equal signals for IL-8. Furthermore, the analysis of mock-conditioned medium did not produce any strong signals (not shown). Two cytokines previously shown to mediate interactions between mesenchymal cells and cancer cells, SDF-1 and CCL5/RANTES, were represented on the array and did not produce detectable signals. To verify the cytokine array data, we performed an ELISA analysis of conditioned media (Figure 6D), focusing on MCP-1, which was a high-abundance cytokine specific to G2B-10A medium. This ELISA analysis showed that G2B-10A medium contained 391 pg/ml MCP-1, while in R2-T1AS medium MCP-1 was undetectable, which validates the array data.

### G2B-10A benign mammary epithelial cells increase the tumorigenicity of R2-T1AS cells *in vivo *independent of factors secreted by benign cells

**Figure 7:**
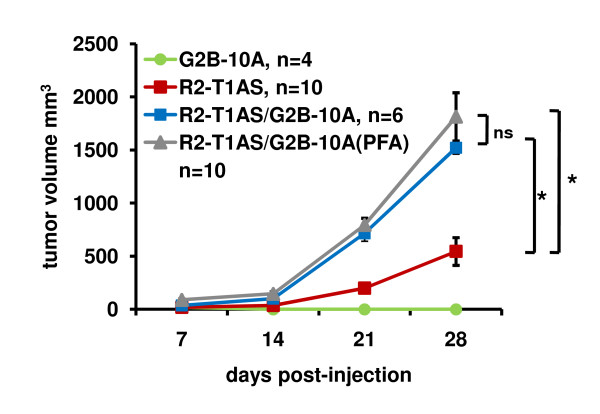
**G2B-10A benign mammary epithelial cells increase the tumorigenicity of R2-T1AS cells *in vivo *independent of benign-cell secreted factors**. Growth of xenograft tumors in nude mice resulting from the injection of 4 × 10^6 ^G2B-10A cells alone (G2B-10A, green line, n = 4), 1 × 10^6 ^R2-T1AS cells alone (R2-T1AS, red line, n = 10), 1 × 10^6 ^R2-T1AS cells mixed with 4 × 10^6 ^G2B-10A cells (R2-T1AS/G2B-10A, blue line, n = 6) and 1 × 10^6 ^R2-T1AS mixed with 4 × 10^6 ^PFA-fixed G2B-10A cells (R2-T1AS/G2B-10A(PFA), gray line, n = 10). Tumor size was measured every 7 days using a digital caliper and tumor volume was calculated using the formula 0.52 × length × width^2^. Data shown are derived from 2 independent experiments. *p < 0.05.

The studies included thus far address the role of benign mammary epithelial cells in enhancing the *in vitro *transformed phenotype of R2-T1AS breast cancer cells. To determine whether benign mammary epithelial cells also influence the phenotype of breast cancer cells *in vivo*, we examined the effects of G2B-10A cells on the tumorigenicity of R2-T1AS cells in a nude mouse xenograft model. First, we injected 4 × 10^6 ^G2B-10A cells alone to verify that these cells were not tumorigenic, and indeed, G2B-10A cells did not form any tumors (Figure 7, green line). To establish the ability of R2-T1AS cells to form tumors in mice, we injected 1 × 10^6 ^R2-T1AS cells alone, which formed tumors that on average measured 545 mm^3 ^in volume on day 28 after inoculation (Figure 7, red line). To determine whether G2B-10A cells influenced the tumorigenicity of R2-T1AS breast cancer cells, we co-injected 4 × 10^6 ^G2B-10A cells with1 × 10^6 ^R2-T1AS cells, maintaining the increased ratio of benign to malignant cells, as used in the *in vitro *studies. As expected from *in vitro *results, the volume of these R2-T1AS/G2B-10A mixed tumors reached 1521 mm^3 ^on day 28, which is about 3-fold larger than R2-T1AS-only tumors (Figure 7, blue line). The data described here are derived from 2 separate experiments. We also performed 3 additional separate experiments (not shown), which yielded similar results. The result shown in Figure 7 recapitulated the *in vitro *studies, in which the G2B-10A cells enhanced the transformed phenotype of R2-T1AS. Next, we tested the hypothesis that the enhanced tumorigenicity of R2-T1AS *in vivo *is mediated by soluble factors secreted by G2B-10A cells, as the *in vitro *data suggest (Figure 3). To this end, we co-injected 1 × 10^6 ^R2-T1AS cells with 4 × 10^6 ^paraformaldehyde-fixed G2B-10A(PFA) cells. Unexpectedly, fixation of G2B-10A cells with PFA failed to reduce the promotion of tumor growth, as the mixed R2-T1AS/G2B-10A(PFA) grafts reached 1813 mm^3^, which is equivalent to the R2-T1AS/G2B-10A mixed tumors. While this result is not fully consistent with what we observed *in vitro*, the complexity of the *in vivo *intact mammary gland may require several influences (secreted factors, direct cell contact, stromal contributions, etc) to promote optimal growth of R2-T1AS tumors in the nude mouse xenograft model.

**Figure 8:**
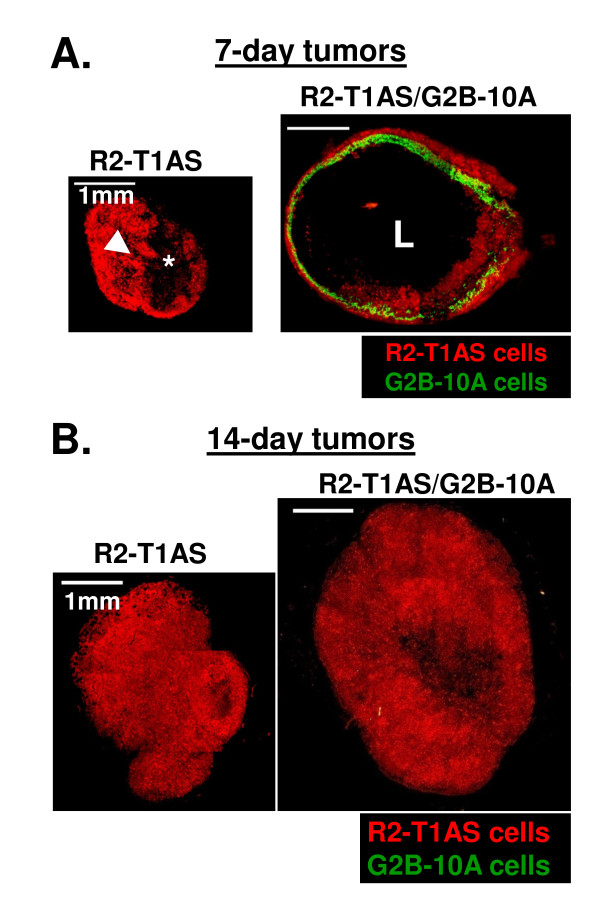
**Morphology and cellular composition of tumors harvested at day 7 and day 14**. (A) Fluorescent sections of tumors harvested at 7 days post-inoculation (red fluorescence - R2-T1AS cells; green fluorescence - G2B-10A cells). Left panel: R2-T1AS - tumors resulting from the injection of R2-T1AS cells alone, partially composed of solid tissue (arrowhead) and partially of necrotic areas (asterisk). Right panel: R2-T1AS/G2B-10A - tumors resulting from the injection of R2-T1AS cells mixed with G2B-10A. Majority (8 of 10) of tumors at day 7 had cystic structure. The lumen of the cyst (L) was filled with fluid and cellular debris, and surrounded by G2B-10A (green fluorescence) and R2-T1AS (red fluorescence) cells. (B) Fluorescent sections of tumors harvested at 14 days post-inoculation (red fluorescence - R2-T1AS cells; green fluorescence - G2B-10A cells). Both R2-T1AS and R2-T1AS/G2B-10A mixed tumors were composed of solid tissue formed by R2-T1AS cells (red fluorescence). Scale bars: 1 mm.

To determine whether G2B-10A cells contribute to tumor masses, we harvested tumors from R2-T1AS/G2B-10A and R2-T1AS groups at 7 and 14 days post-inoculation and we also harvested tumors from all 3 groups (R2-T1AS/G2B-10A, R2-T1AS/G2B-10A(PFA) and R2-T1AS) at 28 days post-inoculation. Tumor sections were analyzed by fluorescence microscopy. As shown in Figure 8A, in the R2-T1AS-only group, the tumors 7 days post-injection were composed of solid tissue (arrowhead), and of necrotic areas of disaggregated cells and debris (asterisk). In the R2-T1AS/G2B-10A mixed group, 8 out of 10 tumors analyzed formed a cyst at 7 days, with the lumen filled with cellular debris and fluid, surrounded by green-marked G2B-10A cells and red-marked R2-T1AS cells. As shown in Figure 8B, at day 14 post-injection, the R2-T1AS/G2B-10A mixed tumors had filled the cyst and developed a solid structure, resulting in tumors larger than the R2-T1AS-only tumors. However, the benign G2B-10A cells were no longer detectable at 14 days. Similarly, at 28 days post-inoculation, tumors from all groups (R2-T1AS, R2-T1AS/G2B-10A and R2-T1AS/G2B-10A(PFA)) were composed solely of R2-T1AS cells forming solid masses, with tumors arising from R2-T1AS/G2B-10A injections and R2-T1AS/G2B-10A(PFA) injections showing no differences (see Additional file 1). This analysis reveals that G2B-10A benign cells disappeared from the tumor tissue within 2 weeks after inoculation. Nonetheless, the early and transient presence of G2B-10A (± PFA) cells resulted in higher tumorigenicity of R2-T1AS cells.

### Both live and PFA-fixed benign G2B-10A cells stimulate R2-T1AS cells to secrete soluble factors

**Figure 9:**
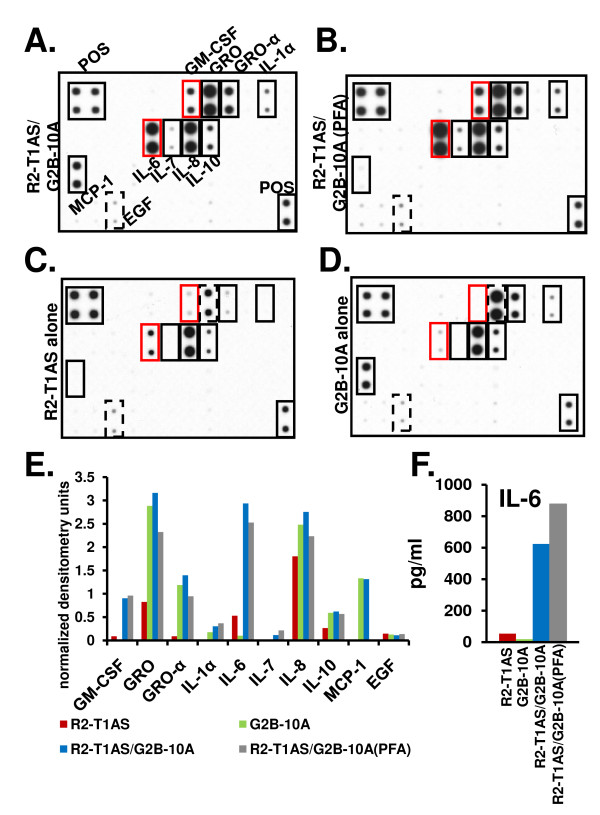
**Both live and PFA-fixed benign G2B-10A cells stimulate R2-T1AS cells to secrete soluble factors**. (A-D) Cytokine antibody array analyses of conditioned media derived from: (A) R2-T1AS/G2B-10A co-culture (1.875 × 10^5 ^R2-T1AS cells/ml mixed with 1.5 × 10^6 ^G2B-10A cells/ml), (B) R2-T1AS/G2B-10A(PFA) co-culture (1.875 × 10^5 ^R2-T1AS cells/ml mixed with 1.5 × 10^6 ^PFA-fixed G2B-10A cells/ml), (C) R2-T1AS alone (1.875 × 10^5 ^R2-T1AS cells/ml). The R2-T1AS cells were plated at 8 times lower density than G2B-10A cells to reflect the 1:8 ratio at which cancer and benign cells were co-cultured in the soft agar assays. (D) G2B-10A alone (1.5 × 10^6 ^cells/ml). To produce conditioned media, cells were cultured in suspension, in low-glucose, 5% serum conditions and conditioned media were harvested after 3 days of incubation. All antibody array membranes were incubated with 1 ml of conditioned media from the indicated cultures. All membranes underwent the same treatment and were exposed to the X-ray film for the same time. Membranes shown were generated in a different experiment than the membranes in Figure 6. POS - positive control spots, black solid lines - differentially expressed cytokines, red lines - signals specific to co-cultures, dashed black lines - not reproducible signals. (E) Quantification of the arrays in Figures 9A, 9B, 9C, and 9D. The signals on the R2-T1AS array (red bars), G2B-10A array (green bars), R2-T1AS/G2B-10A array (blue bars) and R2-T1AS/G2B-10A(PFA) array (gray bars) and were quantified by densitometry and normalized to the average of positive control signals (POS). (F) ELISA analysis of IL-6 concentration in the conditioned media from the following cultures: R2-T1AS (red bars), G2B-10A (green bars), R2-T1AS/G2B-10A (blue bars) and R2-T1AS/G2B-10A(PFA) (gray bars).

To explain the divergence between the *in vitro *result, where the enhanced clonogenicity of R2-T1AS breast cancer cells was mediated by factors secreted by the benign cells, and the *in vivo *result, where G2B-10A benign cells increased the tumorigenicity of R2-T1AS independent of secreted factors, we reasoned that G2B-10A cells may enhance the tumorigenic growth of R2-T1AS cells through direct cell-cell contact, whereby physical contact between R2-T1AS cells and G2B-10A cells (either live, or PFA-fixed) would induce secretion of auto-stimulatory factors by R2-T1AS cells. Such cell-cell contact occurs between R2-T1AS and G2B-10A cells (or R2-T1AS and G2B-10A(PFA cells)), when mixed cells are injected into mice, but not in the soft agar assays. We addressed this hypothesis indirectly, by using cytokine antibody arrays and ELISA assays to analyze conditioned media from the R2-T1AS/G2B-10A and the R2-T1AS/G2B-10A(PFA) co-cultures, in which cells were plated in suspension at a high density, thus allowing them to aggregate and form direct cell-cell contacts (Figures 9A &9B). The conditioned media from these high-density co-cultures were compared to conditioned media derived from either R2-T1AS or G2B-10A cells cultured alone (Figures 9C &9D) and the arrays were quantified by densitometry (Figure 9E). Both R2-T1AS/G2B-10A and R2-T1AS/G2B-10(PFA) co-cultures generated several cytokine signals that were specifically increased compared to R2-T1AS and G2B-10A mono-cultures (Figures 9A &9B). That is, both co-cultures generated a strong IL-6 signal that was increased about 5-fold compared to R2-T1AS alone and weakly detectable in G2B-10A mono-culture. Additionally, both co-cultures also generated a GM-CSF signal that was increased about 10-fold compared to R2-T1AS alone and undetectable in G2B-10A conditioned medium. Also, both co-cultures generated a weak IL-7 signal, which was not generated by G2B-10A or R2-T1AS cells alone. Furthermore, R2-T1AS/G2B-10A and R2-T1AS/G2B-10(PFA) co-cultures generated signals that were selectively increased in both co-cultures compared to R2-T1AS mono-culture (GRO, GRO-α, IL-1α, IL-8 and IL-10), but these factors were also secreted by G2B-10A cells cultured alone. EGF was detected in all cultures, but this signal was not reproducible. To confirm the array data, we performed ELISA analysis focusing on IL-6, which was the cytokine that generated a strong signal specific to the co-cultures (Figures 9A, B, C, D and 9E). The ELISA data showed that the G2B-10A and R2-T1AS mono-cultures contained 19 pg/ml and 54 pg/ml of IL-6, respectively (Figure 9F). Whereas the R2-T1AS/G2B-10A co-culture contained 624 pg/ml IL-6 (12-fold increase over R2-T1AS mono-culture) and the R2-T1AS/G2B-10A(PFA) co-culture contained 878 pg/ml IL-6 (16-fold increase). Analysis of separately prepared conditioned media revealed a similar 4-10-fold increase of IL-6 concentration in R2-T1AS/G2B-10A and R2-T1AS/G2B-10A(PFA) co-cultures compared to the R2-T1AS mono-culture (data not shown). Thus, the strong IL-6 and GM-CSF signals are unique to the co-cultures. Since PFA-fixed cells do not produce active secretions (Figure 3), this result identifies the R2-T1AS cells as the cell type that was induced to secrete IL-6 and GM-CSF cytokines in both co-cultures with G2B-10A (±PFA) cells. This experiment suggests that physical contact between R2-T1AS cells and either live or PFA-fixed G2B-10A cells, resulting in induction of auto-stimulatory cytokines by R2-T1AS cells, is a plausible mechanism responsible for increased tumorigenicity of R2-T1AS cells *in vivo*.

## Discussion

Previous research on the role of the microenvironment in cancer has primarily focused on the interactions between cancer cells and stromal cell populations, rather than interactions between cancer cells and benign epithelial cells. Given that, in breast cancer, the earliest stage tumor cells interact with mammary epithelial cells, and not stromal cells, we used *in vitro *and *in vivo *approaches to show that several cell line models of benign mammary epithelial cells promote the transformed phenotype of R2-T1AS breast cancer cells. While the *in vitro *studies established that factors secreted into the media by G2B-10A cells are required for the enhanced clonogenic behavior of R2-T1AS cells, to our surprise, we found that PFA-treated, metabolically inert G2B-10A cells were able to increase tumor growth *in vivo *as efficiently as non-PFA-treated G2B-10A cells. This result prompted us to determine that PFA-treated G2B-10A cells were able to engage R2-T1AS cells by direct cell-cell contact and cause them to secrete growth factors, such as IL-6 and GM-CSF. In sum, these data are significant because they provide a paradigm shift in our understanding of the role of benign mammary epithelial cells during the course of breast cancer development. Benign MECs are not simply a passive, noncontributory component of the tumor microenvironment, but rather our data highlight that they play a direct role in enhancing the tumorigenic phenotype of breast cancer cells.

In the few previous studies analyzing the effects of benign epithelial cells on the transformed phenotype of distinct cancer cell types, investigators have reported both stimulatory and inhibitory effects [21-26]. With regards to motility and invasion, several studies reported that benign epithelial cells increased the *in vitro *motility and invasion of cancer cells, with breast cancer cell invasion being dependent on SDF-1 secreted by benign epithelial cells [21,24]. In contrast, with regards to cell proliferation, it has been reported that conditioned media derived from benign mammary epithelial cells suppressed proliferation of a variety of breast cancer cell lines in monolayer cultures, in three-dimensional matrigel cultures, in cultures on collagen, and also in soft agar assays [22,23,25,26]. Cumulatively, these studies suggest an inhibitory effect of benign mammary epithelial cells on the proliferation of tumor cells. However, these studies did not address the effects of benign cells on the clonogenicity *in vitro *or tumorigenicity *in vivo *of breast cancer cells.

Our work concentrated specifically on the ability of G2B-10A, G-12A and HuMEC benign cells to enhance R2-T1AS colony formation when cultured at clonogenic (single-cell) density *in vitro *and the ability of G2B-10A cells to enhance R2-T1AS tumor formation *in vivo*, and we consistently found a promotional effect of the benign MECs on these aspects of the transformed phenotype of R2-T1AS breast cancer cells. The relative strength of the effects of the three benign cell lines on the R2-T1AS cells varied between the soft agar and the clonogenicity assays. These differences likely stem from the fact that these assays test for different properties of R2-T1AS cells. Furthermore, under our experimental conditions, HuMEC cells died sooner than G2B-10A or G-12A cells and thus the exposure of R2-T1AS cells to HuMEC cells in the co-cultures was shorter than exposure to the remaining two benign cell lines. Nevertheless, our proliferation data (Figure 4) are consistent with previous reports demonstrating an anti-proliferative effect of benign cells on malignant cells. This anti-proliferative effect of benign cells suggests that the increased R2-T1AS colony numbers and enhanced tumor growth that we observe in response to G2B-10A cells are unlikely to be due to increased R2-T1AS cellular proliferation. In this regard, the results of the clonogenicity assays provided important mechanistic insights. Specifically, we used low-glucose and 5%-serum conditions, such that in these conditions R2-T1AS breast cancer cells exhibited limited capacity for clonogenic growth. However, conditioned media derived from benign mammary cell lines all enhanced R2-T1AS colony numbers 5-days after plating (Figure 5), and since we observed more R2-T1AS colonies with media from benign cells, rather than larger colonies, these data suggest that the key effect is via initial survival. Furthermore, both in soft agar co-cultures and in xenograft assays, the benign cells died early, suggesting again, that their effect is on the initial survival of R2-T1AS cells.

In order to better define the clonogenicity-enhancing activity contributed by benign MECs, we used several *in vitro *approaches and found consistent results showing that G2B-10A cells secrete factor(s) that promote clonogenicity, and that G2B-10A cells do not need to interact with R2-T1AS cells to secrete these factors. Similar *in vitro *studies, employing conditioned media derived from stromal cells, have shown that stromal cells secrete SDF-1 and CCL5, which promote cancer cell proliferation and invasion [12,14,15]. Our array analysis revealed that MCP-1 and IL-1α were secreted by benign G2B-10A cells, but not by malignant R2-T1AS cells, and GRO-α signal was increased in G2B-10A conditioned medium. Of note, both SDF-1 and CCL5 were present on the array, but we failed to detect these two factors in conditioned media derived from either the G2B-10A or R2-T1AS cells. While we have not yet identified the G2B-10A-derived factor contributing to the *in vitro *effects, we proved that secreted factors are key effectors *in vitro*, by treating G2B-10A cells with PFA, which consistently induced the loss of colony formation-enhancing activity contributed by benign MECs in soft agar (Figure 3B). However, the fact that PFA-treated G2B-10A cells promoted equivalent tumor growth as untreated G2B-10A cells *in vivo*, revealed that factors secreted by G2B-10A cells were not required in the *in vivo *assay, and that more complex mechanisms were likely. For example, the *in vivo *xenograft assay tests for several complex features of cancer cells, such as survival in hypoxic conditions, survival in nutrient-deprived conditions, and the ability to establish a vascular network, none of which are factors in the *in vitro *assays.

We propose that benign mammary epithelial cells enhance the tumorigenicity of breast cancer cells *in vivo *by engaging them in direct cell-cell contact between these two cell types, leading to secretion of soluble factors by R2-T1AS that enhance their tumorigenicity. This concept is supported by the results of the high-density co-culture, which allowed direct cell-cell contacts between benign and malignant cells, and showed that G2B-10A cells ± PFA induce a significant increase in secretion of IL-6 and GM-CSF in R2-T1AS cells (Figure 9). A similar effect, whereby direct cell-cell contact between breast cancer cells and mesenchymal stem cells induced secretion of CCL5 by the latter cells has been reported previously [14]. Also, direct contact between cancer cells and serum-activated fibroblasts has been found to stimulate the clonogenic growth of the former [35]. Notably, a recent publication implicated IL-6 as a critical regulator of tumor stem cell renewal, by showing that IL-6 treatment promoted growth of mammospheres formed by MCF-7 cells and primary breast cancer cells [36]. Furthermore, IL-6 and its downstream signaling pathway have been implicated in the regulation of proliferation, survival, and metabolism of cancer cells [37,38]. Finally, normal mammary epithelial cells have been shown to be essential for maintaining and directing the activity of mammary stem/progenitor cells in mammary gland reconstitution studies [39-41]. Taken together, these data imply that cell-cell contact between nontransformed G2B-10A cells and malignant R2-T1AS cells promotes secretion of IL-6 by R2-T1AS breast cancer cells, and that IL-6 is a good candidate to mediate enhanced tumorigenicity of R2-T1AS cells, possibly by inducing tumor stem cell survival.

While our data support the hypothesis that benign MECs increase the tumorigenicity of breast cancer cells by engaging them in direct cell-cell contact leading to secretion of soluble autocrine factors by R2-T1AS, the promotional effects of benign MECs may be explained by several mechanisms. The most obvious possibility is that benign cells increase the tumorigenicity of R2-T1AS via immune cells recruited to the tumor site by G2B-10A cells ± PFA. However, we were unable to establish that immune infiltrates were increased when R2-T1AS cells were injected with the benign cells compared to R2-T1AS cells alone (data not shown). Another possibility is that co-injected benign cells serve as a nutrient source for the breast cancer cells, thus increasing their tumorigenic growth. Indeed, although phagocytosis is not the primary function of mammary epithelial cells, they may in fact phagocytize other cells [42,43]. Another possibility is that the benign cells provide a structural or scaffolding support, whereby the cystic structure observed in tumors 7 days post-injection provides a scaffold that influences oxygenation and/or nutrient availability, which then facilitates tumorigenic growth. However, previously published tumor xenograft studies in nude mice revealed that interactions between cancer cells and activated stromal cells resulted in larger tumors, compared to cancer cells plus normal nonactivated stromal cells, and that this response was mediated by paracrine factors, thus minimizing the contribution of scaffolding effects [13,14]. Finally, we recognize that the PFA-fixed cells may elicit specific, PFA-dependent effects that are separate from those elicited by live G2B-10A cells. Thus, the possibility remains that in mixed R2-T1AS/G2B-10A xenografts, the increased tumorigenicity is in fact dependent on the factors secreted by benign cells, whereas in R2-T1AS/G2B-10A(PFA) xenografts, the effect is dependent on a different, unknown mechanism.

## Conclusions

The critical contribution of this work is that benign MECs directly enhance the clonogenicity and tumorigenicity of breast cancer cells. This is an important and clinically relevant new observation that may play a critical role in the very early stages of cancer development, when a few cancer cells are surrounded by an excess number of adjacent benign epithelial cells. Thus, these results expand our understanding of the tumor microenvironment to include the benign epithelial cell compartment as a relevant contributor to tumor progression.

## Abbreviations

TRIIβ: (TGF receptor IIβ); PFA: (paraformaldehyde); PBS: (phosphate buffered saline); DCIS: (ductal carcinoma in situ); EGF: (epidermal growth factor); GFP: (green fluorescent protein); MEC: (mammary epithelial cell)

## Competing interests

The authors declare that they have no competing interests.

## Authors' contributions

JMP designed the experiments and performed soft agar assays, clonogenicity assays, cell proliferation assays, analysis of tumor sections and cytokine arrays, and she participated in nude mouse tumorigenicity assays. She also wrote the manuscript. JT performed the nude mouse tumorigenicity assays. XL performed comprehensive statistical analysis of the data. PJS provided the MDA-MB-231-T1AS breast cancer cell line and many critical discussions about the concept of the study experimental designs. AGH directed the overall design of the study and participated in the preparation of the manuscript. All authors read, assisted in revision, and approved the final manuscript.

## Pre-publication history

The pre-publication history for this paper can be accessed here:

http://www.biomedcentral.com/1471-2407/10/373/prepub

## Supplementary Material

Additional file 1 **Figure Supplemental 1**. Morphology and cellular composition of tumors harvested at day 28. Click here for file
