# Supplementary material for: Benign mammary epithelial cells enhance the transformed phenotype of human breast cancer cells
Source: BMC Cancer. 2010 Jul 16;10:373. doi: 10.1186/1471-2407-10-373 (PMC2913961; doi:10.1186/1471-2407-10-373)
Supplement: Additional file 1 — Figure Supplemental 1. Morphology and cellular composition of tumors harvested at day 28. [file 1471-2407-10-373-S1.PDF]

28-day tumors

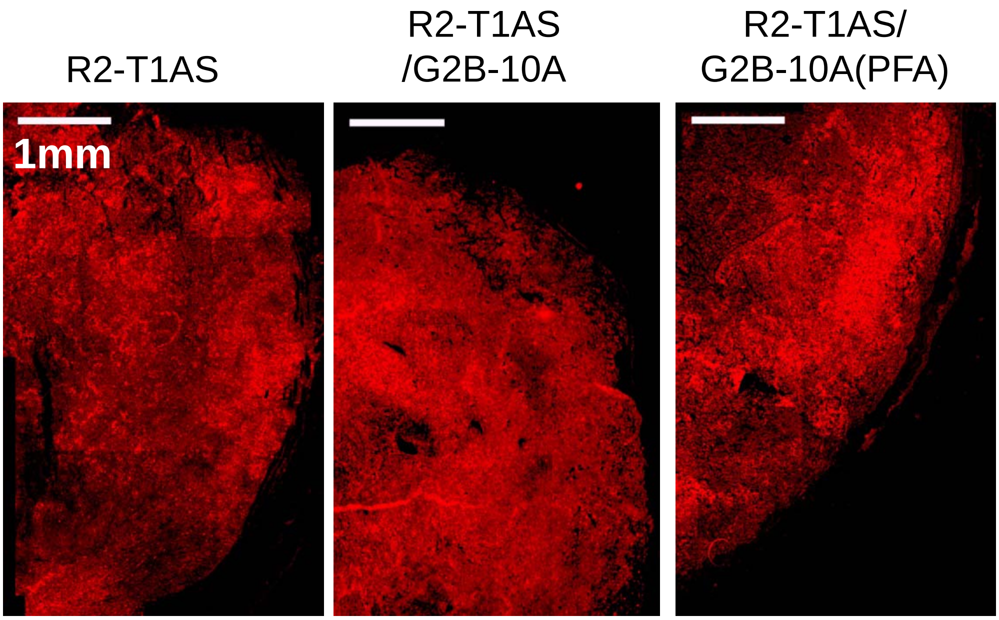

Figure Supplemental 1. Morphology and cellular composition of tumors harvested at day 28. Fluorescent sections of tumors harvested at 28 days post-inoculation (red - R2-T1AS cells). Tumors from all groups (R2-T1AS, R2-T1AS/G2B-10A, R2-T1AS/G2B-10A(PFA)) were composed of solid tissue formed by R2-T1AS cells (red). Images show a representative part of tumor section. Scale bars: 1 mm.
